# True Molecular Composites: Unusual Structure and Properties of PDMS-MQ Resin Blends

**DOI:** 10.3390/polym15010048

**Published:** 2022-12-22

**Authors:** Artem V. Bakirov, Sergey V. Krasheninnikov, Maxim A. Shcherbina, Ivan B. Meshkov, Aleksandra A. Kalinina, Vadim V. Gorodov, Elena A. Tatarinova, Aziz M. Muzafarov, Sergey N. Chvalun

**Affiliations:** 1 Enikolopov Institute of Synthetic Polymer Materials RAS, 70 Profsoyuznaya Str., 117393 Moscow, Russia; 2 National Research Centre “Kurchatov Institute”, 1 Kurchatov sq., 123098 Moscow, Russia; 3 A.N. Nesmeyanov Institute of Organoelement Compounds of Russia Academy of Sciences, 28 Vavilova Str., 119991 Moscow, Russia

**Keywords:** poly(dimethyl siloxane)-rubber molecular composites, SAXS, MQ-resins, mechanical properties

## Abstract

Poly(dimethyl siloxane)-MQ rubber molecular composites are easy to prepare, as it does not require a heterophase mixing of ingredients. They are characterized by perfect homogeneity, so they are very promising as rubber materials with controllable functional characteristics. The manuscript reveals that MQ resin particles can significantly, more than by two orders of magnitude, enhance the mechanical properties of poly(dimethyl siloxane), and, as fillers, they are not inferior to aerosils. In the produced materials, MQ particles play a role of the molecular entanglements, so rubber molecular weight and MQ filler concentration are the parameters determining the structure and properties of such composites. Moreover, a need for a saturation of the reactive groups and minimization of the surface energy of MQ particles also determine the size and distribution of the filler at different filler rates. An unusual correlation of the concentration of MQ component and the interparticle spacing was revealed. Based on the extraordinary mechanical properties and structure features, a model of the structure poly(dimethyl siloxane)-rubber molecular composites and of its evolution in the process of stretching, was proposed.

## 1. Introduction

The development of scientific principles for the creation and modification of elastomers with adjustable molecular structure (molecular weight, composition, stereoregularity) and mechanical characteristics is one of the most fundamental problems of modern materials science. One of the most commercially attractive strategies in the achievement of such a goal is the creation of one general synthetic route, in which variation composition allows the adjustment of the material properties in a substantially wide range. For instance, recent achievements in the chemistry of polyolefines was stipulated by the progress in the design of new catalytic systems [1,2,3,4,5,6,7], which allows the synthesis of polymers with highly defined molecular structure, molecular weight distribution, and correspondingly with a well-defined supramolecular organization, the degree of crystallinity and a wide range of adjustable properties: from rubber-like elastomers to highly crystalline thermoplastics [8,9]. 

Another important class of the elastomeric materials is polysiloxanes (“silicones”), semi-inorganic polymers of the general structure –[SiRR’–O–]n with various alkyl or aryl R and R’ groups. Such polymers have attracted considerable scientific and commercial interest [10,11,12,13] due to many unusual properties, such as good thermal stability, drastically low viscosity-temperature coefficients, substantially high permeabilities, low surface tension, good dielectric properties, weather resistance, attractive release and lubrication properties, high biocompatibility, and excellent optical clarity. Classic, and the most studied and produced silicone, is poly(dimethyl siloxane) (PDMS) –[Si(CH3)2–O–]n. As in all poly(siloxanes), however, it has relatively poor mechanical properties [14,15], substantially limiting the range of its practical use. 

Therefore, to take full advantage of the cited attractive properties in most of their possible applications, poly(dimethyl siloxanes) generally requires reinforcing by a filler. The usual industrial processing of such materials involves an energy-intensive ex situ blending of a reinforcing filler, most commonly fumed silica (SiO2), into the PDMS prior to its crosslinking. The other widely used fillers are titania (TiO2) and zirconia (ZrO2). However, for such composites, the issue of correct dispersion of materials is extremely acute, as mineral nano- and microparticles tend to form large aggregates leading to inhomogeneous distribution of the filler and, accordingly, to the low, inhomogeneous, and unpredictable physical and mechanical properties of the material.

So, from the practical point of view, even the more promising approach is in situ preparation of the hybrid nanocomposite using sol-gel technique [16,17,18,19]. Mark used such technique to produce PDMS/silica [20] or PDMS/titania [21] composites by the ‘sequential’ processing—the first step was the already-described formation of an unfilled PDMS network by tetraethoxysiloxane (TEOS) end linking. The next step involved swelling the extracted polymer network in a ceramic precursor, in this case TEOS [22], possibly in the presence of ammonia as catalyst [23]. This was followed by an acid-catalyzed or base-catalyzed hydrolysis, and condensation of the precursor to generate the reinforcing filler in situ. The analysis of the structure of such systems made it possible to reveal the influence of the conditions of the sol-gel process on the structure and properties of the composites. In the latter case, strongly alkaline conditions and a large excess of water produce uniformly dense particles. These particles appear to be clustered into larger units that also appear to be uniformly dense and nonfractal [20,24]. The growth of fillers under basic conditions is directly analogous to the growth of compact particles via the Stober process [23] for the production of colloidal silica. Synthesis of fractally rough particles due to the changes in pH enhances the bonding between phases and improves material toughness: in solution polymerized alkoxides become increasingly ramified as the pH is lowered and as the water/silicon ratio is lowered [25]. A Mooney-Rivlin analysis of the mechanical properties as a function of pH at roughly the same filler concentration [20] revealed that although there is little difference in effectiveness, the mechanical modulus of the composite increases with pH, whereas maximum extensibility decreases. However, detailed structural analysis carried out in [20] is rather uncertain, as authors switch their interpretation of the fractal nature of silica from the developed surface of unique particle to shape of the agglomeration of smooth, uniformly dense particles. Thus, such research is, until now, important and relevant. 

Another important problem arising in mineral-polymer composites is a substantial risk of crack formation on the organic-inorganic interface. Considerable efforts have been made for better understanding of the interactions between filler particles and PDMS matrix [26,27,28,29,30,31,32,33,34]. They revealed a decrease of PDMS segment mobility in the vicinity of the filler and the presence of a layer of immobilized segments at the particle surface. Reduction in the mobility of the PDMS chains was related to a shift, though rather weak, of the material glass temperature to higher values. Such effects substantially enhance the possibility of crack formation. Furthermore, a local increase of the free energy of siloxane fragments lead to the chain expulsion and, respectively, to the aggregation of inorganic particles. 

The aforementioned phenomena limit the level of the effective filler rate in poly(dimethyl siloxane)-oxide composites. An improvement of the cross-linking of the matrix with silica filler particles could be achieved by the use of bioinspired silica [35,36], polyhedral oligomeric silsesquioxane cages with vinyl groups linked to a central siloxane core by hydrosillylation [37] or MQ copolymers, where M is mono-, Q is tetrafunctional silicon units [38,39,40]. It was established [41] that MQ species have multiple chemical junction points in the silicone network, both covalent bonding and potential noncovalent interactions such as van der Waals, electrostatic or hydrogen bonding. Ultimately, the dynamics of polymer-filler interactions and their influence on mechanical and thermal properties is of utmost importance for understanding and predicting the lifetime and aging of silicone components. Given the strong influence these species can have on silicone composites, improved methods to detect and quantify MQ species influence the organization and mechanical properties of polymer matrix are needed. For instance, the phase structures of lightly cross-linked poly(dimethyl siloxane) (PDMS)/MQ silicone polymer blends were analyzed using scanning probe microscopy (SPM) and pulsed ^1^H NMR [42]. The SPM observations demonstrated that these blends had sea/island-type phase structures, in which hard MQ phases were dispersed in a soft matrix. The pulsed ^1^H NMR showed that the soft matrix was not pure PDMS but rather composed of a PDMS-rich phase incorporating dissolved MQ, whereas the dispersed phase was almost pure MQ. The molecular motion of the PDMS-rich matrix was found to become more constrained with increases in the proportion of MQ. The effect of temperature on the NMR T2 relaxation time spectra was assessed and the MQ domains were determined to partially dissolve in the PDMS matrix at higher temperatures and then to precipitate on cooling. 

Such observations supported in many aspects our own studies which have demonstrated [43] that MQ -resins are also very effective as an active filler, significantly enhancing the mechanical properties of vulcanizates, not inferior in efficiency to aerosils, but without requiring the heterophase mixing of ingredients. Of course, such an influence of the molecular filler was expected and even planned. However, what we could not expect, is the level of the influence—the enhancement of mechanical properties reported in this manuscript is up to date unprecedented—in some materials it reached 350%. Moreover, the discovered ordering in the material that does not contain monodisperse ingredients, turned out to be completely unexpected for us. To what extent this phenomenon, not previously observed in such systems, determines their properties, is the main question for further research. Systematic studies of the structure of such systems and its changes in the process of stretching will shed light and increase understanding of the ways to regulate the properties of elastomers.

## 2. Materials and Methods

A wide range of materials differing in concentration, components, and content of functional groups in them were obtained by adding methyl MQ resins with residual hydroxysilyl groups and carboxyl-containing PDMS as cross-linker (PC) to commercially available PDMS premodified with 3-aminopropyltriethoxysilane (PDMS-A and PDMS-E) matrix. The polymer matrixes are chemically identical in all but one parameter—molecular weight. PDMS-A, based on “SKTN A”™, had Mn = 18,500, Mw = 31,500, Mw/Mn = 1.7 containing 0.14 wt.% of amino groups. A mixture of “SKTN A”™ brand PDMS (138 g, 0.006 mol) and 3-aminopropyltriethoxysilane (2.66 g, 0.012 mol) was stirred at 100 °C for 8 h, then vacuumed at 50 °C and 1 mm Hg to give 140 g of the product (yield 99%). The content of amino groups determined by titration (by 0.1N HCl) was 0.14 wt.%. PDMS-E, based on ”SKTN E”™, had Mn = 72,200, Mw = 120,000, Mw/Mn = 1.7 and 0.04 wt.% of amino groups was synthesized similarly to PDMS-A. The content of amino groups determined by titration was 0.04 wt.%. Detailed synthesis for the used components was described elsewhere [43].

The amount of carboxyl-containing PDMS (cross linker) was 15 wt. parts per 85 wt. parts of PDMS-A and PDMS-E. The amount of MQ was varied in a range from k = 0 to 100 wt. parts per 100 wt. parts of the total PDMS in the formulation. The value of k is presented in our sample notation system: PDMS-X-MQ/k. Here X is either A or E depending on the molecular weight of PDMS. Thus, if k = 40, the mass fraction of MQ will be 40/(100 + 40) = 28.57, and so on.

Films were obtained by pouring a solution containing the required amount of components in methyl tert-butyl ether (MTBE) onto a cellophane substrate. All MQ-based composites, regardless of the composition, rapidly cured at room temperature to form transparent elastomers. The formation of dense films is rather fast after the components are mixed, depending on the composition. Annealing of the films was performed for two hours at 200 °C.

**Figure 1:**
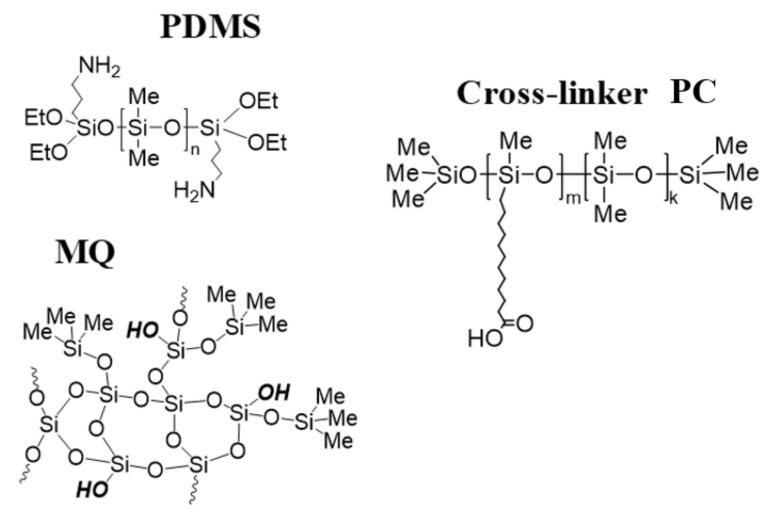
Schematic representations of the constituent components of molecular composites.

In brief, we replaced the preformed silica filler with an MQ resin with Si-OH groups as a molecular filler and crosslinker PC for poly(dimethyl siloxanes) having 3-aminopropyldietoxysilyl groups on the ends. The amine groups were introduced to catalyze the hydrolysis-condensation of ethoxysilane groups. In addition, a polysiloxane blocked on the ends with trimethylsilyl groups and having a certain carboxyalkyl group content on the chain is added as a crosslinker (Figure 1). The cross-linking mechanism was studied in detail and established elsewhere [43]. Networks are formed with mixed covalent and supramolecular or, after annealing, covalent and ionic crosslinks. The presence of such covalent links allows us to state that true molecular composites have been indeed synthesized. 

Compositions of the produced molecular composites, are summarized in the Table 1. 

The mechanical properties of molecular composites were studied before and after the annealing (200 °C for 2 h) in the uniaxial tension mode. A universal testing machine Instron-5965, equipped with a load cell of ±50 N was employed. Mechanical tests were carried out in two modes: single stretching up to a fracture and cyclic loading (100 cycles) in the range of (5 ÷ 50)% (second cycle starts at 5% strain and goes up to 50% of the fracture strain) followed by stretching to fracture. The initial shear rate was 0.1 s^−1^ (60 mm/min). The samples were tested in the dumble-bell shape with the thickness of (0.25 ± 0.02) mm and the size of the rectangular part of 10 × 3.2 mm. Before the experiments, all samples were kept in the air at room temperature for at least 24 h. 

SAXS experiments were carried out at the BIOMUR station of Kurchatov synchrotron (National Research Center “Kurchatov Institute”) using X-ray radiation of 8 keV (wavelength 1.445 Å, resolution dE/E = 10^−3^), photon flux being 10^−9^ s^−1^. The beam spot on samples was (0.3 × 0.5) mm, and the exposure time was chosen to be 600 s. Diffraction patterns were recorded with a Dectris Pilatus 1M detector. Sample to detector distance was approximately 2500 mm, silver behenate standard was used as standard. A simple stretching device was used to study the structure of deformed films. Data processing was performed by Fit2D software. Analysis of distance distribution functions were performed with the use of ATSAS software package [44,45,46,47]. 

The thorough Fourier analysis of the obtained SAXS patterns allows to obtain the additional information on the particle structure in PDMS-MQ resin molecular composites. The interparticle distance was calculated as a position of the first maximum of the Fourier transform of obtained SAXS patterns. The particle size was calculated as a first intersection of the Fourier transform plot with Ox (distance) axis. 

Analysis of the intensity of small-angle X-ray reflections was carried out using two-phase model (MQ particles in PDMS matrix). Here, if the system consists of two phases with different, but constant within each phase, electron densities *η*_1_, *η*_2_, and volume fractions of each phase are *w*_1_ and *w*_2_, respectively. Then the average electron density $\bar{\eta }$ and root-mean-square fluctuation of the electron density 〈Δ*η*^2^〉 is [48]:

(1) $$\bar{\eta }=\eta_{1}w_{1}+\eta_{2}w_{2}\;\langle \Delta \eta^{2}\rangle ={\langle \left(\eta -\bar{\eta }\right)}^{2}\rangle ={\left(\eta_{1}-\eta_{2}\right)}^{2}\cdot w_{1}w_{2}$$

Moreover, root-mean-square fluctuation of the electron density is directly related to the scattering (Porod) invariant:

(2) $$Q=\int_{0}^{\infty }s^{2}I\left(s\right)ds=2\pi^{2}V_{0}\langle \Delta \eta^{2}\rangle$$

where *V*_0_ is an irradiated volume. So:

(3) $$Q=2\pi^{2}V_{0}{\left(\eta_{1}-\eta_{2}\right)}^{2}w_{1}w_{2}$$

As electron density of each phase *η* is determined by its mass density *ρ*

(4) $$\eta =\frac{\sum_{i}Z_{i}}{\sum_{i}M_{i}}\rho$$

where *Z_i_* and *M_i_* are the charge and the mass of an *i*^th^ atom constituting a repeating unit of matter. Thus, for a two-phase system with sharp phase boundaries, the following system of equations takes place:

(5) $$\rho =\rho_{1}c+\rho_{2}\left(1-c\right)\;\langle \Delta \eta^{2}\rangle =\frac{1}{2\pi^{2}V_{0}}\int_{0}^{\infty }s^{2}I\left(s\right)ds={\left(\Delta \eta \right)}^{2}\cdot c\left(1-c\right)$$

Thus, combination of thorough chemical characterization, mechanical tests and X-ray studies allowed to provide a detailed view of the structure of obtained PDMS-MQ molecular composites, as well as of its changes in the process of mechanical deformation. 

## 3. Results

### 3.1. Mechanical Behavior

**Figure 2:**
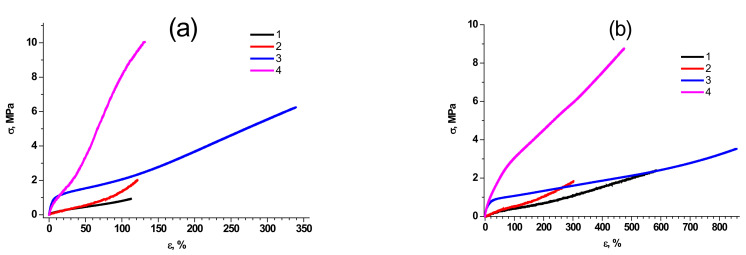
Typical deformation curves of molecular composites based on PDMS-A (**a**) and PDMS-E (**b**), filled with pristine MQ resin PDMS-MQ/10 (1), annealed PDMS-MQ/10 (2), pristine PDMS-MQ/50 (3) and annealed PDMS-MQ/50 (4).

As we have stated in the Introduction, the synthesis of polymers with highly defined properties and molecular structure is possible due to the precise control of polymer molecular characteristics. First of all, we have to emphasize that the mechanical properties of the synthesized molecular composites depend on both the structure of PDMS rubber and the filling rate. Figure 2 reveals typical stress-strain curves of molecular composites based on PDMS of different molecular weight and MQ resins. A complete array of data on the main mechanical properties of the studied composites is given in our previous work [43] and briefly summarized in Table S7. It can be easily concluded that composites based on PDMS-E are characterized by significantly higher elongation at break than those based on PDMS-A at comparable filling (600–850% PDMS-E-MQ composites vs. 100–300% in PDMS-A-MQ) and lower tensile strength—as the chain length of PDMS-A is lower than that of PDMS-E, molecular composites of the former are closer to the extension limit, and their deformation is followed by stronger loss of chain entropy. This observation is true for both pristine and annealed samples. 

The tensile strength of pristine composites significantly (almost by 30 times) increases, regardless of the brand of rubber, with an increase in filling rate, reaching the values of 6.2 MPa in PDMS-A-MQ/50, and 5.9 MPa in PDMS-E-MQ/100 of MQ resin. Such effect can be ascribed to the cross-linking role of MQ particles in the synthesized composites.

**Figure 3:**
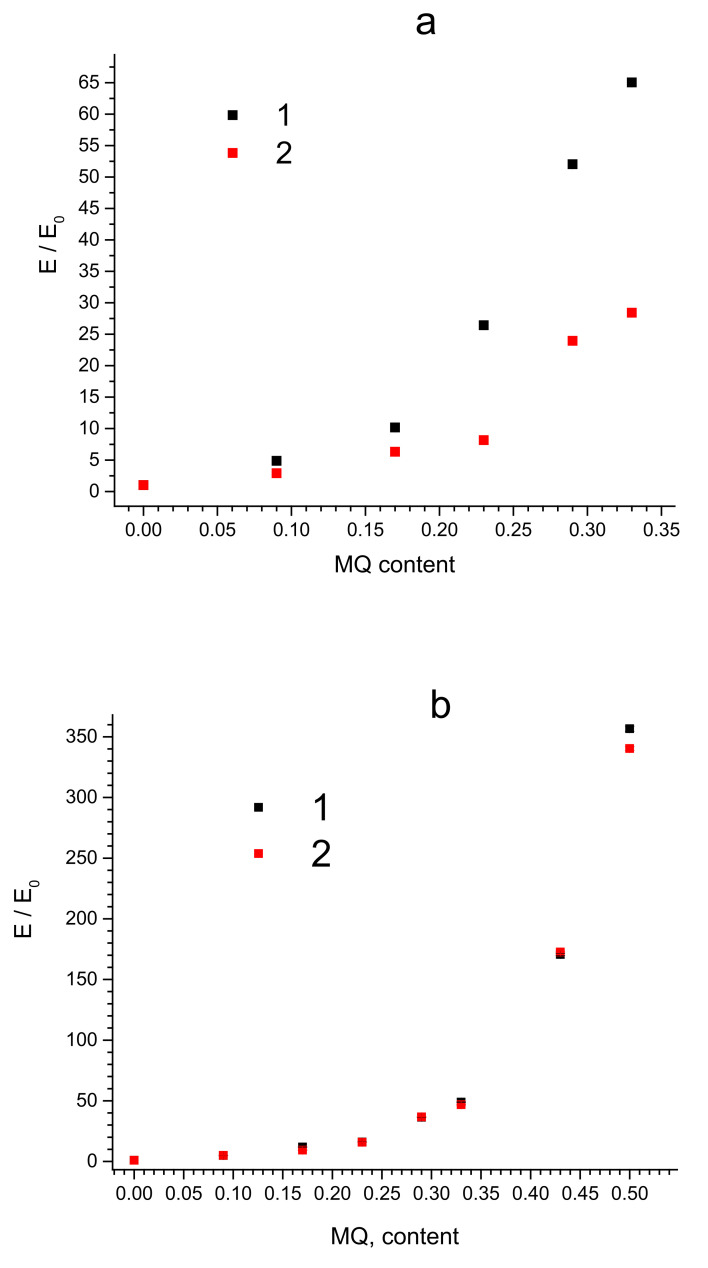
Dependencies of normalized Young modulus ratio E /E_0_ for PDMS-A (**a**) and PDMS-E (**b**) based composites with varied filling content by MQ resin (wt%) pristine (1) and annealed (2).

The tensile modulus (Figure 3) also increases with an increase in the filling rate over the entire range of MQ content in the composites based on both PDMS-A and PDMS-E: its maximum increase was observed for PDMS-A-MQ/50 (16.0 MPa, more than 60 times compared to the pure PDMS-A) and, correspondingly, to PDMS-E-MQ/100 (51.0 MPa, more than 350 times compared to the PDMS-E). The fact that in most cases the increase in mechanical properties occurs rather monotonically with an increase in the degree of filling indicates a good distribution of MQ particles in the rubber matrix. We must also emphasize that such an enormous growth of the mechanical properties is by far absolutely unprecedented, thus making PDMS—MQ true molecular composites commercially promising materials. It seems even more important, as the fillers in the produced composites—MQ resin particles—are isotropic, so the use of composites produced is not as limited as in case of anisotropic fillers, which promise better mechanical enhancement but work in presupposed directions only.

Annealing leads to a decrease in the tensile modulus of molecular composite, which is much more pronounced in PDMS-A based materials compared to those based in PDMS-E. Such fact is also easily explained by the introduced model and by the fact that PDMS-A molecules are ~4 times shorter than those of PDMS-E. Contour length of the average PDMS-A chain is LA = 120 nm, and that of PDMS-E is LE = 480 nm. Assuming the Kuhn segment to be the same in both polymers and equal to 1.56 nm, as it was established by SANS studies of condensed PDMS [49], one could estimate that the coil size would be 13.7 and 27.4 nm in PDMS-A and PDMS-E respectively. Thus, as we show below, the distance between MQ particles corresponds to the average coil size in PDMS-A on low filling rates, while in PDMS-E composites of the same filling rate it is equal to the size of about two coils. On highest filling rates the interparticle distance corresponds 25% of the contour length of PDMS-A and to only 18% of the contour length of PDMS-E. Thus, short PDMS-A chains in the pristine material must be substantially more straightened between the MQ particles than much longer, and, thus, not so strained chains of PDMS-E. The relaxation of such chains is, correspondingly, much more pronounced in the process of annealing of PDMS-A based composites. In parallel, additional curing and rearrangement of siloxane network due to Si-OH back-biting must also be much more pronounced in straightened and short PDMS-A chains exposing all their functional groups.

Tensile strength also increases in composites based on both PDMS-A and PDMS-E. The growth is monotonic up to 33% filling rate of MQ resin, which is close to the practically achievable limit for PDMS-A based systems (up to 10 MPa, or more than 30 times, in the annealed PDMS-A-MQ/50, and 8.6 MPa in PDMS-E-MQ/50). The nature of such growth is due to the cross-linking which includes formation of chemical bonds, as annealing, activating the crosslinker, which polymerizes the matrix, leads to an even greater increase in the material strength. This process does not affect the MQ filler structure at any content. However, with further increase of the filling in PDMS-E based molecular composites tensile strength starts to fall down (6.6 MPa in PDMS-E-MQ/100). 

Undoubtedly, such significant, and often complex, changes in mechanical properties are associated with the evolution of the structural organization of composites at the molecular level during both filling and subsequent heat treatment. Therefore, thorough structural studies are important to analyze such variations in the composites with different MQ content and thermal prehistory.

### 3.2. Small-Angle X-ray Scattering (SAXS)

**Figure 4:**
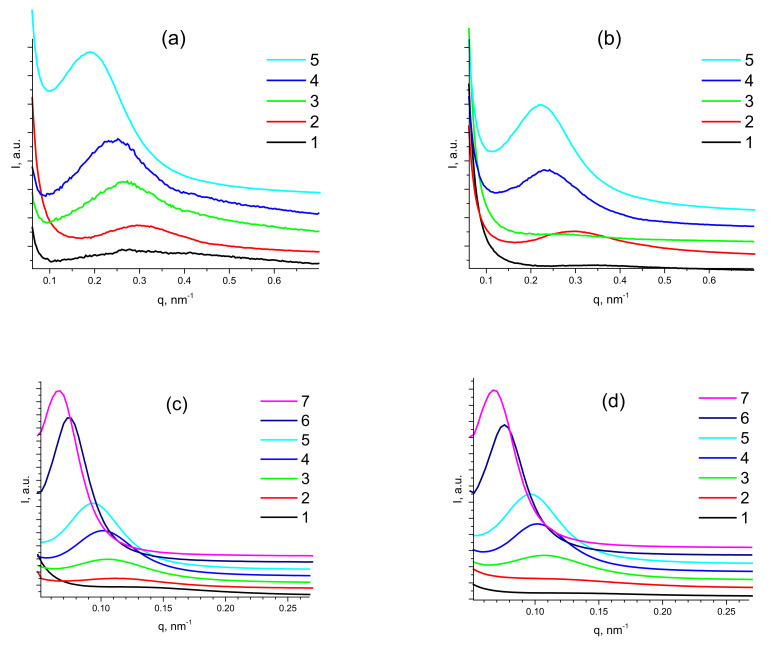
SAXS curves of pristine (**a**) and annealed (**b**) PDMS-A-MQ/10 (1), PDMS-A-MQ/20 (2), PDMS-A-MQ/30 (3), PDMS-A-MQ/40 (4) and PDMS-A-MQ/50 (5). Same for pristine (**c**) and annealed (**d**) PDMS-E-MQ/10 (1), PDMS-E-MQ/20 (2), PDMS-E-MQ/30 (3), PDMS-E-MQ/40 (4), PDMS-E-MQ/50 (5), PDMS-E-MQ/75 (6) and PDMS-E-MQ/100 (7).

With such a goal, USAXS studies were carried out for PDMS-A and PDMS-E films with MQ resin content varied from 9 wt% to 50 wt%. SAXS patterns of pristine (a,c) and annealed (b,d) samples of PDMS-A (a,b) with (9 ÷ 33) wt% of MQ resin filler and PDMS-E (c,d) with (9 ÷ 50) wt% of MQ resin (Figure 4) reveal a distinct Bragg reflection shifting to lower q values with increasing MQ content in both PDMS-A and PDMS-E based composites.

This reflection must be attributed to the relatively uniform distance between MQ resin particles possessing higher electron density compared to the PDMS matrix. This fact is proved by an increase in the reflection intensity with the filling rate (see Figure S1 in the Supporting Information), as it is directly proportional to the contrast in electron density between the filler and matrix (Equation (5) in the Experimental). Moreover, we have carried out thorough Fourier analysis of the obtained SAXS patterns of all PDMS-MQ resin molecular composites (see Figure S2 in the Supporting Information). The interparticle distance also substantially increases with the filling rate: from 15 nm in PDMS-A-MQ/10 to 33 nm in in PDMS-A-MQ/50, and from 43.0 nm in PDMS-E-MQ/10 to 65 nm in PDMS-E-MQ/50 and further to 95 nm in PDMS-E-MQ/75. The particle size calculated as a first intersection of the Fourier transform plot with Ox (distance) axis—size of MQ particles, was proved to increase from 6.0 nm in PDMS-A-MQ/20 to 13.2 nm in in PDMS-A-MQ/50, and from 20.0 nm in PDMS-E-MQ/20 to 26.8 nm in PDMS-E-MQ/50 and further to 37.6 nm in PDMS-E-MQ/75.

Thus, an increase in the filling rate in PDMS-MQ resin molecular composites leads to the growth of distance between the filler particles. Similar results were obtained for annealed samples of both PDMS-A and PDMS-E composites with varied MQ content. 

**Figure 5:**
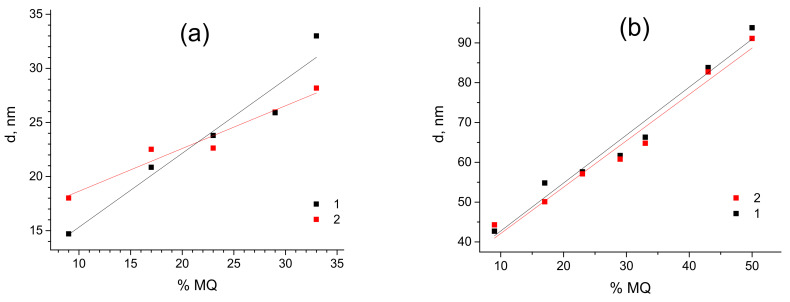
Dependencies of d-spacing in PDMS-A (**a**) and PDMS-E (**b**) based composites with varied filling rate by MQ resin (wt%) pristine (1) and annealed (2).

The fact that the more filler was added to the composite the larger is the distance between the MQ particles, is rather counterintuitive, as one would expect a decrease in such distance upon adding more filler to the composite. However, the described tendency is quite unambiguous (see Figure 5).

**Figure 6:**
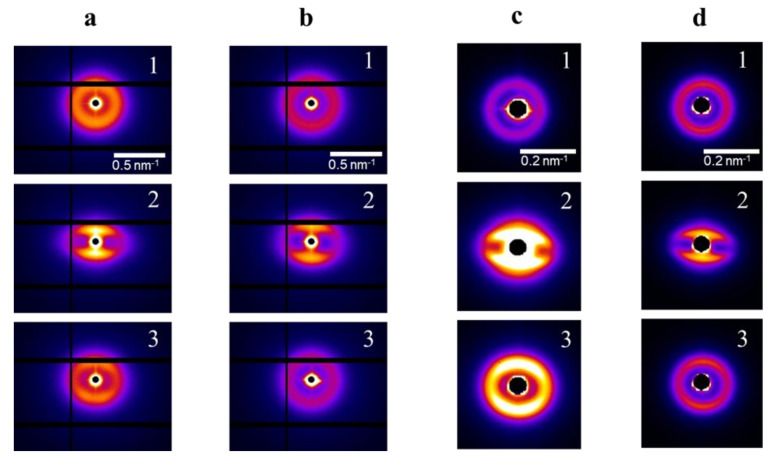
2D SAXS diffraction patterns for pristine (**a**) and annealed (**b**) PDMS-A with 33 wt% MQ, (1) initial nondeformed film λ = 1; (2) deformed to λ = 1.5; (3) restored after deformation. Same for pristine (**c**) and annealed (**d**) PDMS-E with 33 wt% MQ.

Our considerations are also supported by the results of the in-situ SAXS measurements of the uniaxial deformation of the obtained molecular composites. Figure 6 reveals 2D diffraction patterns of initial, stretched to 150% and recovered after the load, pristine and annealed samples of PDMS-A and PDMS-E based composite films, the deformation axis is vertical. As one can see, the isotropic pattern changes upon deformation, the two bright meridional arc reflections shift to lower angles, while equatorial reflection weakens due to a disturbance of structural ordering in lateral direction. The changes in the position of reflections (from 32 to 49 nm for PDMS-A and from 66 to 110 nm for PDMS-E) correlate well with the macroscopic deformation. Actually, one can see that for all the studied samples (see Figure S3, Tables S1 and S2 in the Supporting Information), uniaxial elongations are with a good degree of accuracy equal to the proportional changes of the interparticle distance. Thus, they strongly could be named affine. Further deformation to 200% leads to further transformation of the meridional reflection (they take a «Strich» shape), and to a complete disappearance of equatorial scattering. The text continues here. The total pool of X-ray data is presented in Tables S3–S6 for pristine (Tables S3 and S5) and annealed (Tables S4 and S6) samples of PDMS-MQ composites based on PDMS-A (Tables S3 and S4) and PDMS-E (Tables S5 and S6).

We must also emphasize that thermal annealing does affect the reversibility of structural changes in the sample after restoring its original shape. Residual deformation of pristine samples is substantial, manifesting itself in the anisotropic SAXS pattern, while annealing, activating the cross linker and, correspondingly, making the molecular entanglement network denser, led to the virtually full recovery to the original SAXS pattern.

### 3.3. Cyclic Deformation and Hysteresis

**Figure 7:**
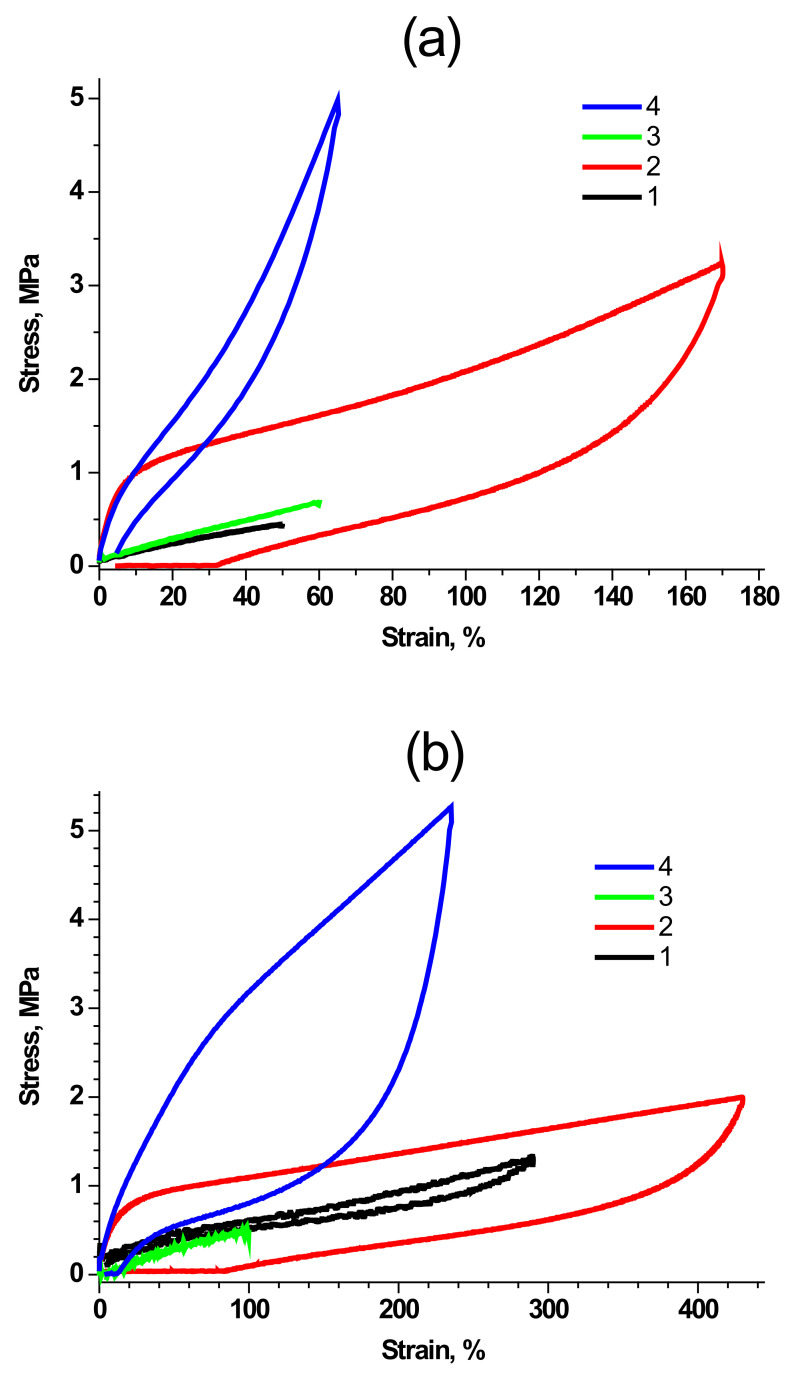
(**a**) Cyclic deformation (hysteresis) of pristine PDMS-A-MQ/10 (1), PDMS-A-MQ/50 (2) and annealed PDMS-A-MQ/10 (3) and PDMS-A-MQ/50 (4). (**b**) Same for pristine PDMS-E-MQ/10 (1), PDMS-E-MQ/50 (2) and annealed PDMS-E-MQ/10 (3) and PDMS-E -MQ/50 (4). First cycles are shown.

An important question arises about the nature of the links between PDMS matrix and MQ rubber particles. Are they reversible? Can MQ particle and polymer coil rearrange in the process of deformation. To reveal the nature of such links in the produced materials, we performed a series of tests on cyclic loading of the studied samples in a universal testing machine. Figure 7 represents mechanical hysteresis loops for samples of pristine (a) and annealed (b) PDMS-A with 9 wt% (1, PDMS-A-MQ/10) and 33 wt% (2, PDMS-A-MQ/50), as well as for pristine (c) and annealed (d) PDMS-E with 9 wt% (1, PDMS-E-MQ/10) and 33 wt% (2, PDMS-E-MQ/50). It is clearly seen that in the materials with the low MQ filling (PDMS-A-MQ/10 and PDMS-E-MQ/10) deformation and stress are completely reversible in the loading-unloading cycle, and the differences are only observed in the hysteresis value (area of the hysteresis loop). Before heat treatment, the observed hysteresis area is much larger for the sample based on PDMS-E; after heat treatment, we did not observe a noticeable effect of the polymer matrix on the mechanical hysteresis at this MQ content. We observe much more differences (both in size and shape of the loop) when filling silicone rubbers containing with 33 mass parts of MQ resin. First of all, attention is drawn to the presence in non-heat-treated samples of significant residual deformation, which reaches almost 90% for a sample based on PDMS-E. This permanent deformation completely disappears after heat treatment in the sample based on PDMS-A and is significantly reduced (up to about 15%) in the material based on PDMS-E. It also completely changes the shape of the deformation curve in the unloading, which acquires a pronounced “S” shape, with a sharp (instead of smooth) decrease in stress in the sample at the last (less than 50% deformation) stage of unloading. This behavior is probably associated with both the distribution of particles (or aggregates) of the MQ resin in the polymer matrix, and with the specific interaction of this active filler with silicone rubbers.

### 3.4. Molecular Model of the PDMS-MQ Composites

**Figure 8:**
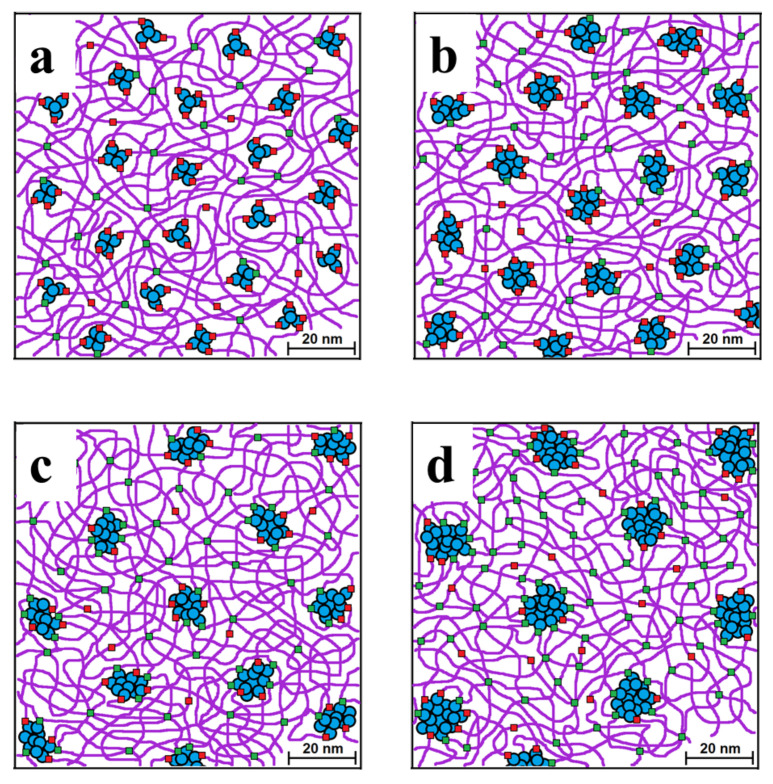
Model of packing of PDMS-A with 17 wt% (**a**, PDMS-A-MQ/20) and 33 wt% (**b**, PDMS-A-MQ/50). Same for PDMS-E with 17 wt% (**c**, PDMS-E-MQ/20) and 33 wt% (**d**, PDMS-E-MQ/50). Contour length: PDMS-A 120 nm, PDMS-E 480 nm.

Figure 8 represents a model illustrating our understanding of the ordering process in the studied polymer composition. As we well know, MQ copolymer is a composite material, individual fractions of which are molecular colloidal particles (high molecular weight fraction with a rigid core–blue balls). Low molecular weight fractions contain a minimum amount of functional groups and play the role of a plasticizer, which can be evenly distributed throughout the body of the composition (red balls). The middle fraction has a greater number of functions and is more mobile than the high molecular weight fraction and plays the role of a polyfunctional branching center. Finally, the green balls are physical knots of topological and electrostatic interactions (hydrogen bonds of carboxyl groups with each other or with hydroxyl groups). The model adequately describes the aggregation of molecular colloids (blue) around the nodes formed at the initial stage and explains the increase in the distance between nodes, which is controlled by the long linear PDMS molecules used in the process of composite formation.

MQ particle size increases with increasing filling rate. This observation is rather obvious as the more filling material is present in the polymer matrix, the easier it is for its particles to find each other and form a larger aggregate. Moreover, the higher the concentration of MQ particles in the mixture, the shorter the distance between them, and, thus, the stronger the polymer expulsion forces. Such model had already been proposed for this type of composites, but on another dimension scale and filling degree [50].

The more interesting is the comparison between PDMS-A and PDMS-E composites with the same filling rates (compare Figure 8a,c, and Figure 8b,d). One can see that resin particles in PDMS-E based composites are substantially larger. This can be explained by the larger molecular weight of PDMS-E which correspondingly has lower concentration of reactive end groups compared to PDMS-A. So, reactive groups of MQ particles have lower chances of binding to the polymer matrix and must be saturated by the binding to a resin particle minimizing the free surface energy of the particle and of the material as a whole. Such a need for a saturation of the reactive groups and minimization of the surface energy determines also an increase of the distance between the particles with increasing filler rate–smaller particles migrate to the larger ones forming large aggregates, which are far one of another.

## 4. Conclusions

In this manuscript we have demonstrated that the manipulation of the synthesis conditions of molecular MQ-PDMS composites allows the precise adjustment of their structure and mechanical behavior. Between the main instruments of such control is the size and distribution of the MQ particles playing a role of polymer network junctions. The formation of such particles is determined by several factors: the aggregation of nanoparticles due to polymer depletion forces; the minimization of interface surface; the saturation of the chemical interaction of functional groups of the MQ particles and of the PDMS matrix; and the entropic elasticity of a polymer chain, determined by its length. It is important to note that from a common point of view such materials as cross-linked networks have no long-range order and, thus, it is very difficult to characterize them using scattering methods. In this aspect, the presence of a distinct X-ray reflection in the prepared materials and, correspondingly, their structural ordering, is a very important, though not unique observation.

Previously, we have demonstrated [43] that MQ copolymers exhibit their molecular nature by working as a homogeneous crosslinking agent; the produced materials are transparent, and their AFM scans present the perfectly uniform distribution of MQ particles in PDMS. Our current study adds to that argument; observed X-ray reflection is possible only for the systems with a very uniform distances between the hard particles in the matrix. Moreover, MQ copolymers are very efficient as active fillers. These fillers significantly enhance the mechanical properties of vulcanized rubber; they are not inferior to aerosils but do not require a heterophase mixing of ingredients. The variation of rubber type, MQ filler concentration and mechanical stress affected strongly the SAXS scattering. While the sample with blocked hydroxyl or without MQ filler showed no Bragg reflection, all other composites showed a distinct maximum, unlike any aerosol based composite materials. This structural information was also enriched by applying the mechanical stress, which led to further increase of distance between filler particles in the meridional direction and to vanishing of the equatorial component. The observed strong growth of the mechanical properties on filling is by far an unprecedented thus making PDMS–MQ true molecular composites commercially promising materials.

The annealing affected on the number of cross-links manifesting itself in the reversibility behavior in cyclic deformation studies. Therefore, such molecular composites with perfect homogeneity are very promising as rubbers with controllable functional characteristics.

## Figures and Tables

**Table 1:** Compositions of molecular composites obtained.

Sample	Content in Composites, Mass%			
	PDMS-A	PDMS-E	MQ	PC
PDMS-A-MQ/10	77.27	-	9.09	13.64
PDMS-A-MQ/20	70.83	-	16.67	12.5
PDMS-A-MQ/30	65.38	-	23.08	11.54
PDMS-A-MQ/40	60.72	-	28.57	10.71
PDMS-A-MQ/50	56.67	-	33.33	10
PDMS-E-MQ/10	-	77.27	9.09	13.64
PDMS-E-MQ/20	-	70.83	16.67	12.5
PDMS-E-MQ/30	-	65.38	23.08	11.54
PDMS-E-MQ/40	-	60.72	28.57	10.71
PDMS-E-MQ/50	-	56.67	33.33	10
PDMS-E-MQ/75	-	48.57	42.86	8.57
PDMS-E-MQ/100	-	42.5	50.00	7.5

## Data Availability

Not applicable.

## Supplementary Materials

The following supporting information can be downloaded at: https://www.mdpi.com/article/10.3390/polym15010048/s1, Figure S1: Dependence of integral intensity of the SAXS Bragg peak on the c*(1-c); Figure S2: Distance distribution function of PDMS-A-MQ/20 and PDMS-A-MQ/50 (a) and PDMS-E-MQ/20, PDMS-E-MQ/50 and PDMS-E-MQ/100 (b); Figure S3: Affinity plots for PDMS-A (a), PDMS-E pristine (b) and annealed (c) samples; Table S1: PDMS A—d-spacing upon deformation, nm; Table S2: PDMS E—d-spacings upon deformation, nm; Figure S4: SAXS curves for PDMS-A-MQ/3.5 and PDMS-A-MQ/50 (a) and PDMS-A-MQ/50 but blocked hydroxyl groups, preventing interaction between the matrix and the filler(b); Table S3: 2D SAXS images for pristine PDMS-A with varied MQ content upon deformation and following restoration; Table S4: 2D SAXS images for annealed PDMS-A with varied MQ content upon deformation and following restoration; Table S5: 2D SAXS images for pristine PDMS-E with varied MQ content upon deformation and following restoration; Table S6: 2D SAXS images for annealed PDMS-E with varied MQ content upon deformation and following restoration; Table S7: Mechanical properties of PDMS-A and PDMS-E based composites before and after annealing.
