# Supplementary material for: True Molecular Composites: Unusual Structure and Properties of PDMS-MQ Resin Blends
Source: Polymers (Basel). 2022 Dec 22;15(1):48. doi: 10.3390/polym15010048 (PMC9823799; doi:10.3390/polym15010048)
Supplement: Supplementary file 1 [file polymers-15-00048-s001.zip › polymers-2067862-supplementary.pdf]

Supporting information for paper

True molecular composites: Unusual ordering phenomenon in blended of PDMS - MQ rubbers

Bakirov A.V., Krasheninnikov S.V., Shcherbina M.A., Meshkov I.B., Kalinina A.A., Gorodov V.V., Tatarinova E.A., Muzafarov A.M., Chvalun S.N.

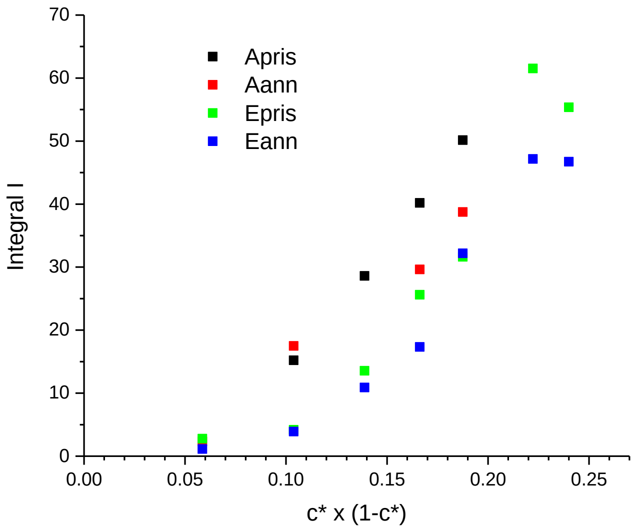

Figure S1. Dependence of integral intensity of the SAXS Bragg peak on the c*(1-c), where c* is the volume MQ concentration. The linear dependence at higher MQ concentrations means that most of MQ particles participate in knot formation.

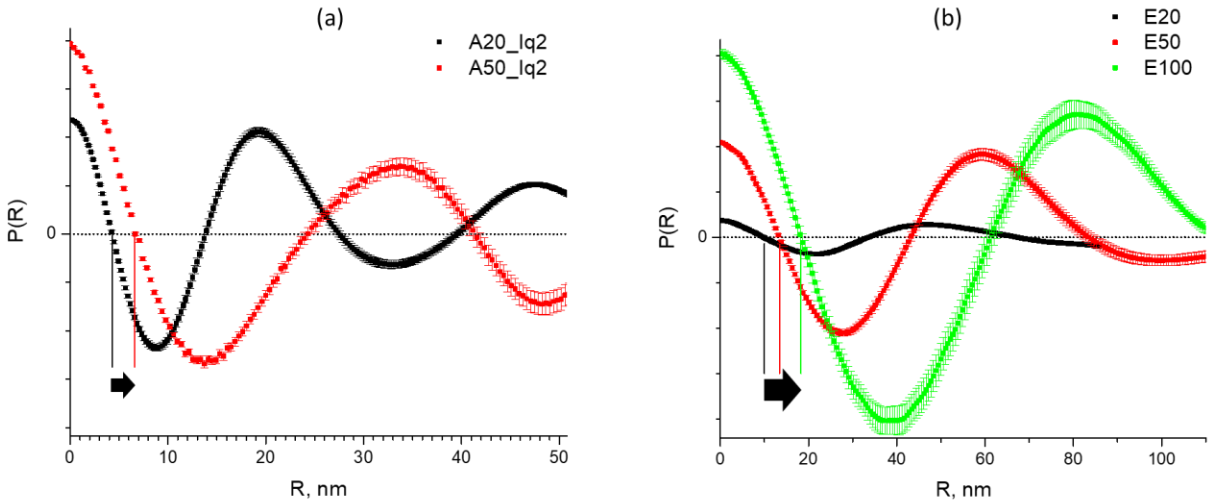

Figure S2. Distance distribution function of PDMS-A-MQ/20 and PDMS-A-MQ/50 (a) and PDMS-E-MQ/20, PDMS-E-MQ/50 and PDMS-E-MQ/100 (b). Black arrows indicate the shifting of zero intersection of correlation functions on increasing of the MQ content. Therefore, the size of the scattering cluster (node) increases from 3 to 6.6 nm for PDMS-A and from 10 to 13.4 and further to 18.3 nm for PDMS-E.

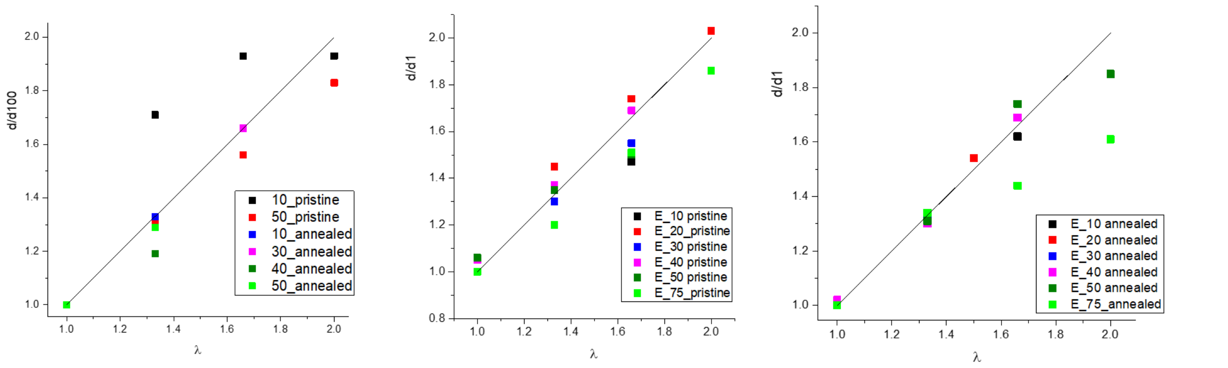

Figure S3. Affinity plots for PDMS-A (a), PDMS-E pristine (b) and annealed (c) samples. Black line indicates the fully affine deformation.

Table S1. PDMS A - d-spacing upon deformation, nm

sample	annealed	λi/λ0 = 1	1.33	1.66	2	relaxed
PDMS-A-MQ/10	-	14.7	25.2	28.3	28.3	24.1
PDMS-A-MQ/10	+	18.0				
PDMS-A-MQ/20	-	20.9				
PDMS-A-MQ/20	+	20.2	26.8			
PDMS-A-MQ/30	-	23.8	30.8	39.4	-	24.1
PDMS-A-MQ/30	+	22.6				
PDMS-A-MQ/40	-	25.9	30.8	-	-	25.9
PDMS-A-MQ/40	+	26.0				
PDMS-A-MQ/50	-	31.5	41.2	49.1	57.6	
PDMS-A-MQ/50	+	27.8	35.8	-		27.8

Table S2. PDMS E – d-spacings upon deformation, nm

sample	annealed	$\lambda_i/\lambda_0 = 1$	1.33	1.66	2	relaxed
PDMS-E-MQ/10	-	42.7	58.0	62.8		42.7
PDMS-E-MQ/10	+	44.3	58.1	71.8		44.3
PDMS-E-MQ/20	-	54.8	79.5	95.4	111.4	56.2
PDMS-E-MQ/20	+	50.1		77.4		
PDMS-E-MQ/30	-	57.6	74.6	89.5		60.9
PDMS-E-MQ/30	+	57.1	75.8	96.6		57.1
PDMS-E-MQ/40	-	61.7	84.8	104.4		64.8
PDMS-E-MQ/40	+	60.8	78.8	102.9		62.3
PDMS-E-MQ/50	-	66.3	89.4	99.1		70.5
PDMS-E-MQ/50	+	65.9	88.1	116.6		68.3
PDMS-E-MQ/50	-	65.8	87.9	116.8	158.7	
PDMS-E-MQ/50	+	64.8	85.0	112.4	119.7	
PDMS-E-MQ/75	-	83.8	100.3	126.4	155.5	93.8
PDMS-E-MQ/75	+	82.7	111.0	156.3	205.2	89.8
PDMS-E-MQ/100	-	93.8	134.8	208.2	257.1	148.7
PDMS-E-MQ/100	+	91.1	146.3	283.7	315.5	130.5

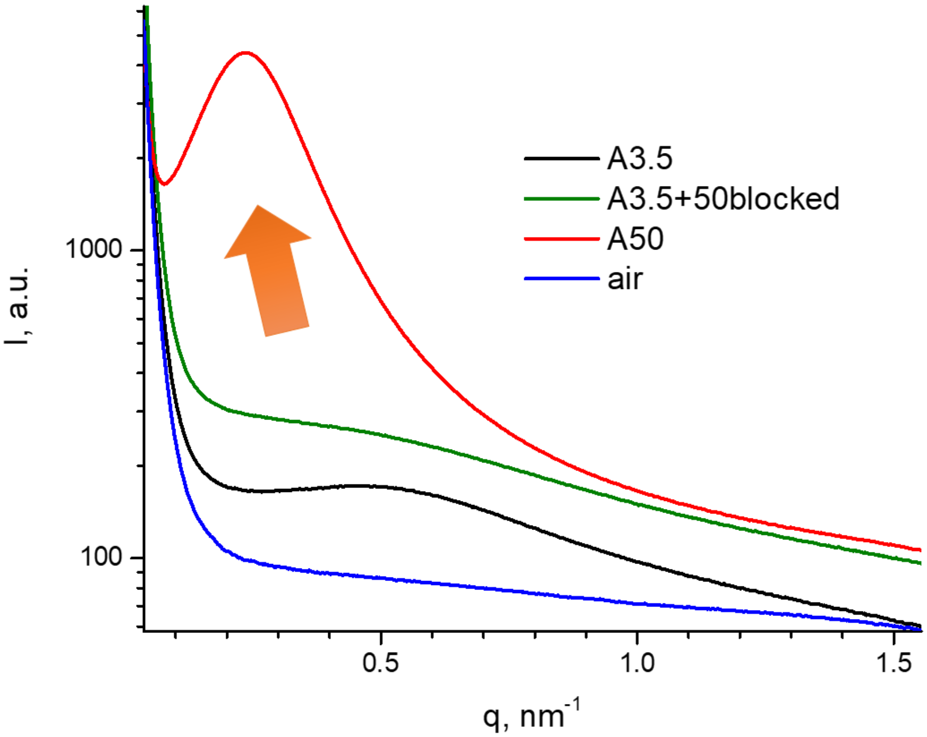

Figure S4. SAXS curves for PDMS-A-MQ/3.5 and PDMS-A-MQ/50 (a) and PDMS-A-MQ/50 but blocked hydroxyl groups, preventing interaction between the matrix and the filler(b). Arrow represents the changes in the intensity in logarithmic scale upon adding this interaction

Table S3. 2D SAXS images for pristine PDMS-A with varied MQ content upon deformation and following restoration.

MQ% content	L = 1	L = 1.33	L = 1.66	L = 2	restored
10	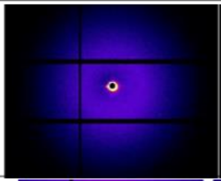	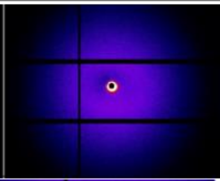	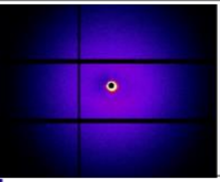	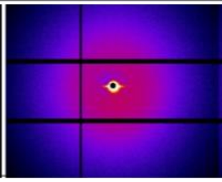	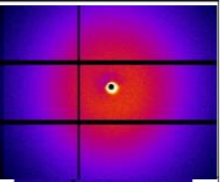
20	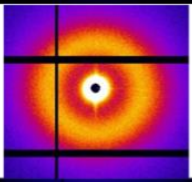	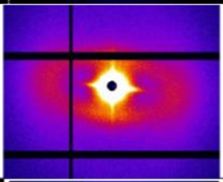	n/a	n/a	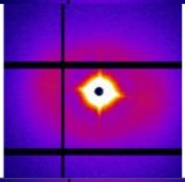
30	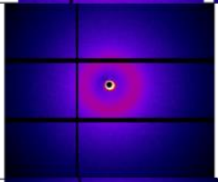	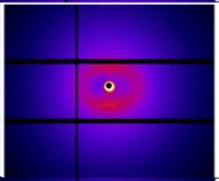	n/a	n/a	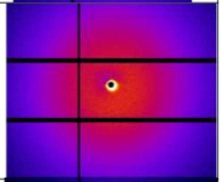
40	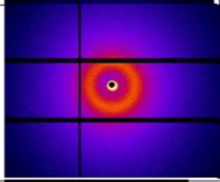	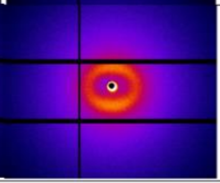	n/a	n/a	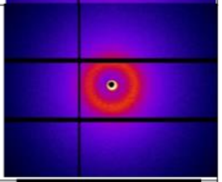
50	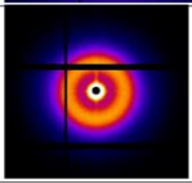	n/a	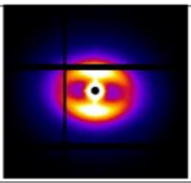	n/a	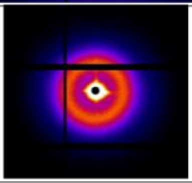

Table S4. 2D SAXS images for annealed PDMS-A with varied MQ content upon deformation and following restoration.

MQ% content	L = 1	L = 1.33	L = 1.66	L = 2	restored
10	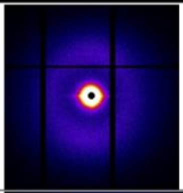	n/a	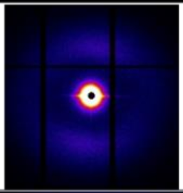	n/a	n/a
20	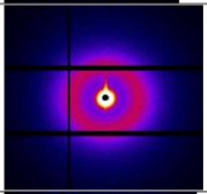	n/a	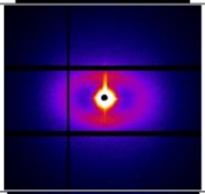	n/a	n/a
30	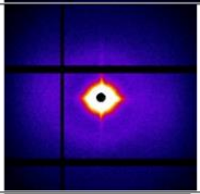	n/a	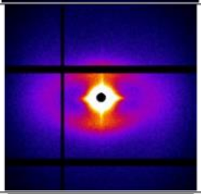	n/a	n/a
40	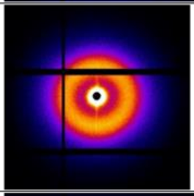	n/a	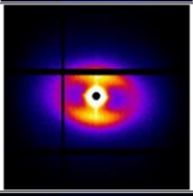	n/a	n/a
50	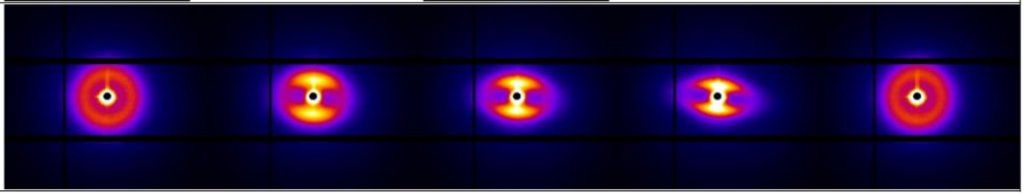				

Table S5. 2D SAXS images for pristine PDMS-E with varied MQ content upon deformation and following restoration. Intensity logarithm was applied to enhance visibility.

MQ% content	L = 1	L = 1.33	L = 1.66	L = 2	restored
10	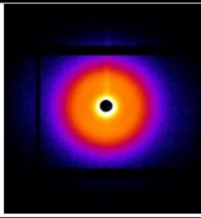	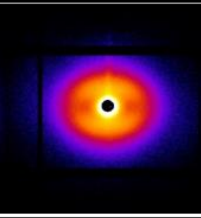	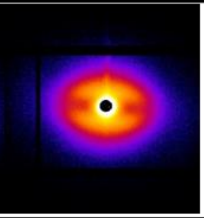	n/a	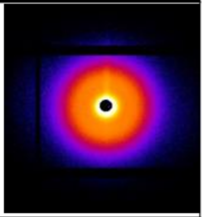
20	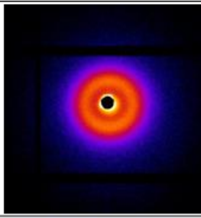	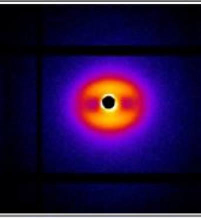	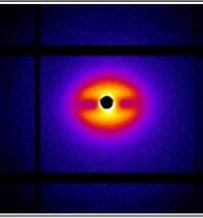	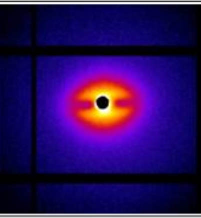	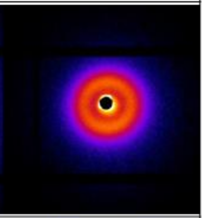
30	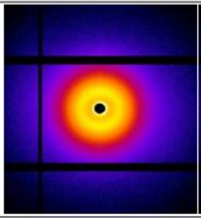	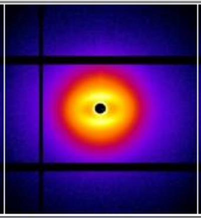	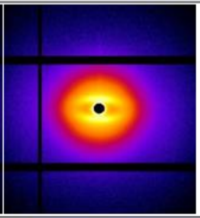	n/a	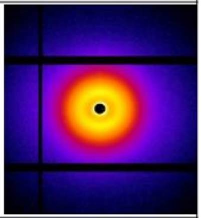
40	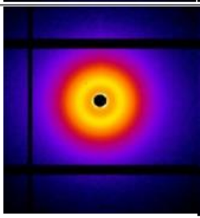	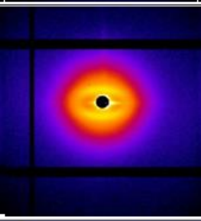	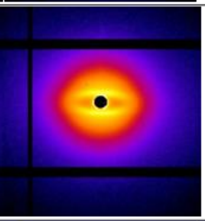	n/a	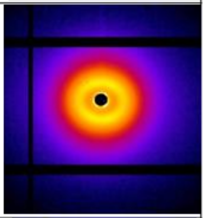
50	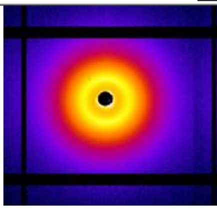	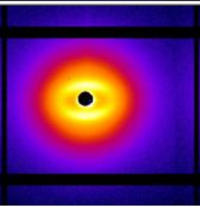	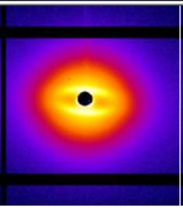	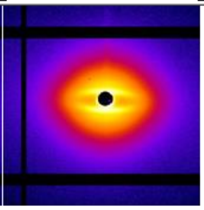	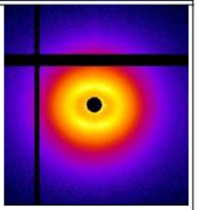
75	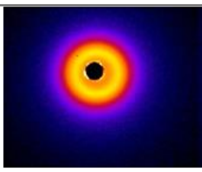	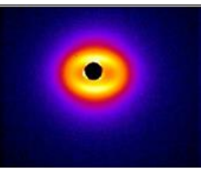	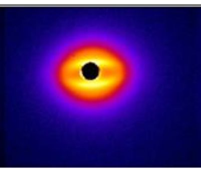	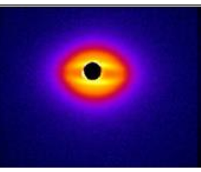	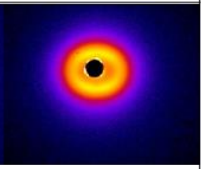
100	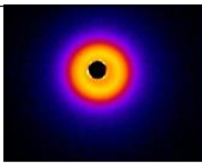	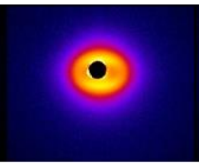	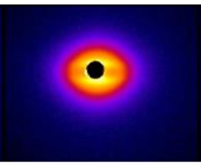	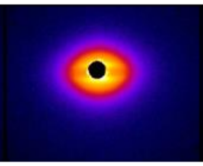	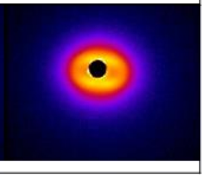

Table S6. 2D SAXS images for annealed PDMS-E with varied MQ content upon deformation and following restoration. Intensity logarithm was applied to enhance visibility.

MQ% content	L = 1	L = 1.33	L = 1.66	L = 2	restored
10	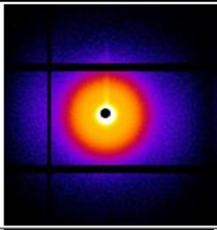	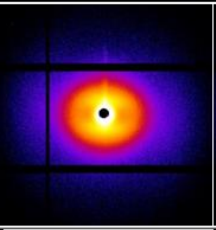	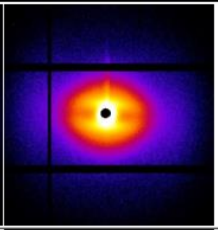	n/a	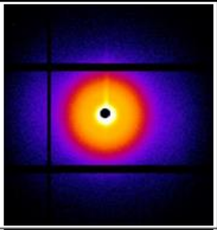
20	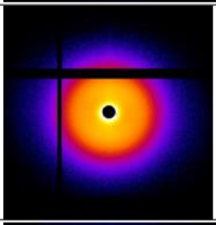	n/a	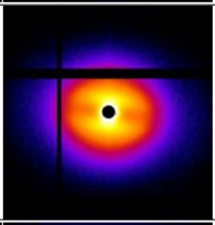	n/a	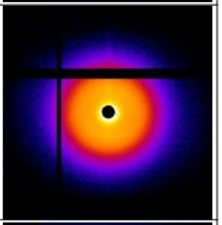
30	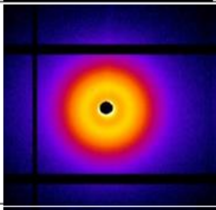	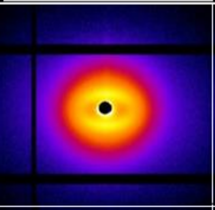	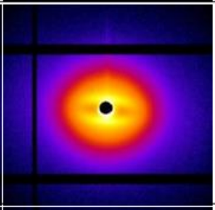	n/a	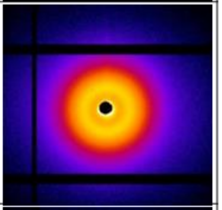
40	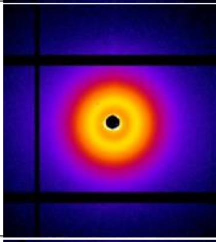	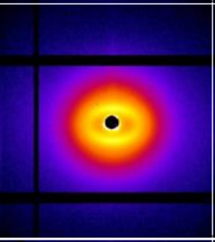	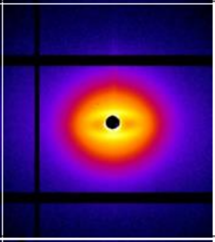	n/a	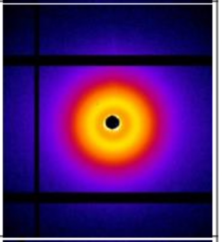
50	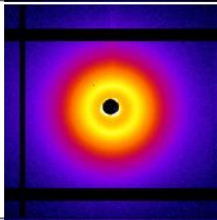	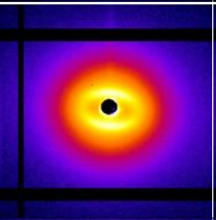	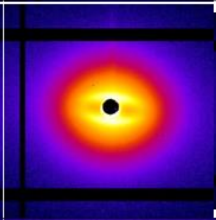	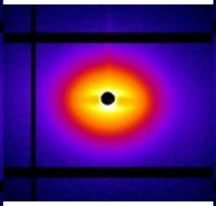	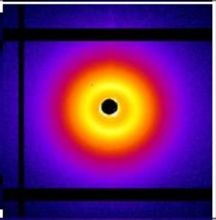
75	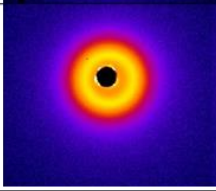	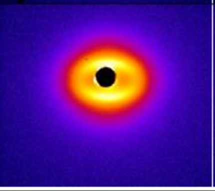	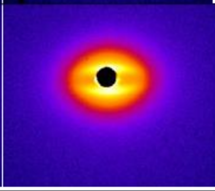	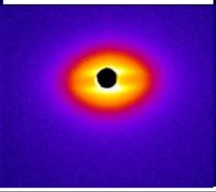	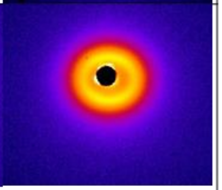
100	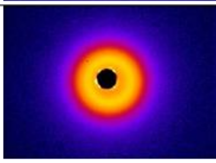	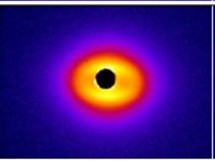	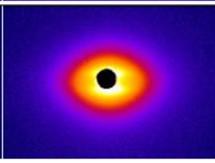	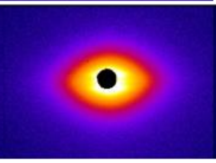	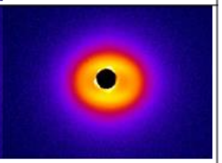

Table S7. Mechanical properties of PDMS-A and PDMS-E based composites before and after annealing.

#	Sample	σ_{max}, MPa		ε_b, %		E, MPa	
		before	after	before	after	before	after
1	PDMS-A-MQ/3.5/50*	0.9±0.1	1.6±0.1	257±16	331±10	0.5±0.05	0.5±0.05
2	PDMS-A-MQ/3.5	0.8±0.1	0.9±0.1	229±11	229±11	0.5±0.04	0.6±0.05
3	PDMS-A-MQ/10	0.9±0.1	2.0±0.1	112±8	121±8	1.2±0.05	1.1±0.04
4	PDMS-A-MQ/20	2.5±0.1	4.5±0.1	194±16	113±7	2.5±0.1	2.4±0.1
5	PDMS-A-MQ/30	4.6±0.2	4.5±0.1	193±12	96±12	6.5±0.2	3.1±0.1
6	PDMS-A-MQ/40	4.9±0.2	7.0±0.3	194±18	101±9	12.8±0.2	9.1±0.2
7	PDMS-A-MQ/50	6.2±0.2	10.0±0.4	339±24	132±11	16.0±0.4	10.8±0.2
8	PDMS-E-MQ/10	2.4±0.2	1.8±0.1	582±32	302±12	0.7±0.1	0.7±0.1
9	PDMS-E-MQ/20	1.9±0.1	4.2±0.2	393±19	378±20	1.7±0.1	1.3±0.1
10	PDMS-E-MQ/30	2.9±0.2	5.8±0.3	498±22	437±14	2.3±0.1	2.2±0.1
11	PDMS-E-MQ/40	3.1±0.2	8.0±0.3	560±24	539±22	5.2±0.2	5.1±0.2
12	PDMS-E-MQ/50	3.5±0.2	8.6±0.2	858±39	453±20	7.0±0.2	6.5±0.2
13	PDMS-E-MQ/75	5.7±0.2	7.2±0.3	575±15	240±11	24.4±0.9	24.0±0.7
14	PDMS-E-MQ/100	5.9±0.3	6.6±0.2	525±19	121±9	51.0±2.1	47.3±1.6
